# Pathology of Insanity

**Published:** 1854-04-01

**Authors:** James George Davey

**Affiliations:** Northwoods, Bristol


### PATHOLOGY OF INSANITY.

*(To the Editor of the Psychological Journal.)*

SIR,—Will you allow me the privilege to offer a few remarks in reply to the strictures of Dr. Hitchman, on certain passages, contained in my "*Nature and Proximate Cause of Insanity?*"

The essay of mine is, it is perceived, divided into two parts; the first designed to show the "*contradictory*" evidence of various medical writers on the subject of which it treats; and the second to prove the correctness of my own peculiar views.

To Dr. Lockhart Robinson I am indebted, solely, for the summary of Dr. Hitchman's opinions, as contained in this gentleman's "*Report on Psychological Medicine,*" published in Vol. xv. of "*Ranking's Abstract of Medical Sciences,*" and NOT to the *Psychological Journal,* nor to the Lectures in the *Lancet,* as is conjectured by your correspondent.

I quoted Dr. Hitchman's words in reference to the morbid condition of the *grey matter* in "*acute sthenic mania,*" in order to contrast them with the very opposite—i. e. "*contradictory*" expressions of CONOLLY and SOLLY. It does seem strange that whilst the colour of the *cineritious neurine* is by the first-named writer said to be of a "*roseate hue,*" the same is declared by the second to be of "*a dark plum colour,*" and by the third as "*generally very pale.*"

So far, Mr. Editor, I am responsible for no "*error,*" or "*distortion,*" or "*misrepresentation;*" and this much Dr. Hitchman will, I doubt not, allow; on *re*consideration.

Your correspondent complains that I have given "no further elucidation of his opinions—except such extracts *as would lead the reader to believe* that *he limited* the physical causes of insanity to a roseate condition of the cerebral hemisphere." Even if this assertion were not an "*error*" I feel that no kind of blame could be, even then, attached to myself, seeing that I was *not* engaged in an exposition of Dr. Hitchman's views on psychological medicine, but rather in the attempt to demonstrate the reasons for additional investigation and farther inquiries into this department of science. However, at page 11, I see that I have quoted the following words from Dr. Hitchman. These have very certainly escaped his attention, because they cannot by any possibility lead the reader of my humble performance to believe that he (Dr. Hitchman) "*limits the physical causes of insanity to a roseate condition of the cerebral hemispheres,*" viz., "*insanity is essentially dependent on some change or irritation produced in the vesicular neurine of the convolutions of the brain; and that malady is influenced by the same laws, and dependent on like physical changes of material structure, as are diseases of the lungs or any other viscera.*"

As Dr. Hitchman has concluded his communication to your journal by placing in direct opposition certain extracts from my "*Nature and Proximate Cause of Insanity,*" and certain other quotations from his own published writings contained in various medical journals, and inasmuch as the similarity of opinions therein conveyed, when taken in conjunction with their respective dates, would seem to imply that *I* had borrowed somewhat largely of his labours and opinions, I may be permitted to add that long ere Dr. Hitchman wrote on *insanity,* I had thus expressed myself. And *my* thanks are due to *Dr. Hitchman* "*for confirming my opinions in language so like my own as the following,*" viz.:—

HITCHMAN.

"I believe that before the scalpel can reveal opacity, thickening, and infiltration of the membranes, or congestion, inflammation, softness, or hardness of the medullary matter, there must have been great and important changes long going on; and that necroscopic appearances ought to be regarded more as *results* than causes—as the *effect* rather than the *source* of the malady."—*Lancet*, vol. ii. 1847, p. 564. (Reprinted in the *Psychological Journal* for October 1st, 1853.)

DAVEY.

"If we imagine an individual labouring under intense *avarice, grief*, or *pride*, it would follow that the increasing physical action of the same portion or portions of the brain would tend to the development of such a state of susceptibility or *irritation* of the part or parts concerned, that, at length, the volition would become suspended; or, in other words, the morbid action would acquire so great a supremacy as to subjugate every other feeling and propensity; and which of course, must be, as above asserted, incompatible with the healthy physical capacities of the cerebral mass. If such an abnormal state remains unrelieved, nothing is more likely than the occurrence of inflammation of the brain and its membranes, more or less insidious, and which progressing would necessarily induce those *palpable* disorganizations of structure, effusions, &c., so commonly observed. Such, I repeat it, are generally the EFFECTS of insanity, and not its first CAUSE."—*Zoist*, vol. i. 1843, p. 116.

Such, Mr. Editor, is my reply to the strictures of Dr. Hitchman.

May I be permitted to make a few observations in reference to *one* remark in your notice of my small book?

The reviewer writes thus:—"*We confess that we are not aware that the theory*" (meaning that one propounded in Dr. Henry Munro's book), "*was distinctly announced by the present author*" (meaning myself) "*in his former publications, although*" (it is added) "*enough was stated to establish, inferentially, a corresponding idea in the mind of the writer; but in a manner as in no way to detract from the claims, such as they are, of the preceding theorist,*" meaning Dr. H. Munro.

The annexed passages from the book of Dr. Henry Munro and those by myself,* if placed in juxta-position, with their respective dates, will directly settle the question of priority; and in asking you for the necessary space in the columns of your journal to prove the point at issue, I trust I may not, under the present circumstances, be considered either egotistical or intrusive:—

MUNRO.

"Insanity is a disease of nervous origin."—p. 79. (1851.)

"The positive symptoms of insanity reveal a greater resemblance to those of *nervous irritation* than to those of acute inflammation of the brain."—p. 85. (1851.)

In insanity "there is no fever, the face is pallid, *the skin damp and cold;* everything about the body giving the indication of anything but inflammatory

* See ZOIST, Vol. i., p. 111, *et seq.*, 1843, "On the Pathology of Insanity," by "*Dr. Davey, of the Hanwell Lunatic Asylum.*" An Essay written with the express object of reconciling the contradictory statements of Jacobi Calmeil, Foville, and others, and of showing upon what grounds the several pathological changes occurring to the brain and its membranes in insanity, and described by these writers, are to be viewed as the mere *effects* of an antecedent *cause*, &c. &c.

action; and yet the mind is in a state of most extravagant delusion."—p. 82. (1851.)

"Therefore, though persons suffering from general debility are a good specimen for showing the asthenic nature of this disease."—p. 70. (1851.)

"But my chief fear is that I may appear to be straining at a gnat, which is most easy to swallow, in writing so much as I have done to fortify my position of the nervous nature of this disease—since everything about it so clearly points out the truth of this position; *its mode of access*, so frequent after excitement, and exhaustion of the nervous system, after paralyzing shocks, after the action of depressing passions, *after drains to the bodily system*, as in puerperal insanity from over-nursing, &c.; again, *its mode of departure*." —p. 71. (1851.)

"The *comprehensiveness* of the theory is one of its greatest advantages; for it can include, equally, the insanity arising from moral shocks, from bodily disease, or from an actual lesion of the brain itself; while, if we rest content with an emotional cause alone, how could we account for the insanity produced on a sudden by a moral shock? and, on the other hand, if we looked to mental causation alone, how could *we account* for that produced evidently by bodily disease or physical injury?"—p. 79. (1851.)

"On the *relation* inflammatory action bears to insanity, my view of this important subject is, that no doubt frequently great congestions, and sometimes inflammatory action in the brain, take place in persons subject to insanity; that when they do so take place, THEY AGGRAVATE THE VIOLENCE OF SYMPTOMS IN ALL CASES; and very probably in many cases the insane paroxysm does not occur until the infirm brain is subjected to this deleterious influence; 2. That this inflammatory action is to be considered of an *asthenic* nature; 3. That inflammatory action can, under no view of the case, be the original cause of insanity; 4. That it cannot be looked upon as a condition essential to insanity; 5. That the presence of inflammation confirms rather than invalidates the theory that insanity is a disease of nervous and vital depression."—p. 81. (1851.)

"Good diet and strengthening medicines will quiet their furor when depletory measures increase it."—p. 115. (1851.)

"All enlightened physicians will prefer the invigorating influence of good diet to any mere theoretic or artificial modes of improving their patients' health. Bitter tonics are often useful."—p. 140. (1851.)

### DAVEY.

"I consider insanity to be essentially a *nervous disease*, and the consequence of an *irritation* of the ultimate structure of the brain—a *neuralgia* of its sensory fibres."—*Zoist*, vol. i. p. 117. (1843.)

"The exceptions to this *rule* are cases consequent on meningeal or cerebral inflammation—whether or not dependent on local injury. What very materially confirms the above position is the fact that the most violent forms of furious mania commonly occur in persons of *weak* and *delicate fibre*, and great susceptibility. I frequently witness the most urgent symptoms of acute insanity in combination with a small and feeble and quick pulse, *cold skin*, and a retracted and anxious countenance."—*Zoist*, vol. i. p. 117. (1843.)

"I cannot help thinking it almost impossible for any medical man well acquainted with the nature and peculiarities of the various forms of insanity, to entertain adverse opinions to those contained in this paper; but so it is.

"They should well remember that attacks of insanity, even recent ones, are, occasionally, not only as sudden in their occurrence as those of *neuralgia*, *hysteria*, &c., but are also no less temporary, and equally severe, comparatively speaking; and, like the last-named diseases, may be either idiopathic or symptomatic. Moreover, it (insanity) is among the effects of a severe hæmorrhage, or *loss of blood;* and is then to be cured only *by a removal of its cause.*

"How could all this happen if it depended on an *inflammation* of any part of the brain or its membranes?"—*Zoist*, vol. i. p. 119. (1843.)

"No one can doubt that every single thought and feeling is associated with certain physical and *molecular* changes in some part or parts of the brain; and, if so, every case of insanity, however slight and temporary, must consist of an abnormal action of a portion of its ultimate structure; and this continuing to increase in intensity and extent so affects the vascular condition of the brain and its membranes that to it at length we become indebted for the more palpable and demonstrable pathological conditions already spoken of. Now the varieties and innumerable modifications of altered structure, as regards locality, &c. &c., are of course no less dissimilar than the several indications of insanity or abnormal cerebration; and therefore we are enabled *to account*, as before mentioned, not only for the *contradictory* opinions already specified, but also for *the association of similar pathological appearances, whether of the brain or membranes, with very opposite manifestations of the disorder.*"—*Zoist*, vol. i. p. 113. (1843.)

"It may be added that the morbid appearances noticed in those who have died of insanity, *for the most part*, hold the same relation to each other that those common to *asthma*, *hooping-cough*, and *angina pectoris* do to these several diseases respectively." . . . "The analogy between the above-mentioned diseases does not end here, for not only are very similar remedial means applicable to them all, both in their complicated and uncomplicated states, but in each one the pathologist not unfrequently verifies the following words of an eminent living writer, viz., '*Changes* may take place in the nervous system not only sufficient to cause the most acute disease, but even to subvert life, without being *so gross* as to be demonstrable to the senses.' If, however, these same '*changes*' are not sufficiently intense to destroy the life of the individual, the chances are they become, eventually, succeeded by others of a very palpable and demonstrable nature, which are not only sufficient in themselves to very seriously impair the healthy function of the part or parts concerned, but existing, as they may be presumed to do, in common with their *first cause*, NECESSARILY AGGRAVATE ALL THE SYMPTOMS OF DISEASE. Among the insane, this precise state of things robs progressively the whole nervous system of its power, and as a consequence every vital function becomes more and more impeded and enfeebled; and the suffering party is left only to vegetate and die."

"The very common indications of the existence of past or present inflammatory action of the brain or membranes, I consider a proof of not only the *occasional* association of the disorder (insanity) with inflammation, as its immediate cause; but also of the *frequent* occurrence of such in the progress of insanity: that is, of that form of disease consequent on "*nervous irritation*."

"The *origin* and *progress* of many cases of insanity, are sufficient to verify this position. Suppose, for the sake of illustration, that an individual of delicate fibre is suddenly frightened by some cause or other, and instead of her recovering from the consequences of alarm, they continue with aggravated severity."

"The faintest sound which reaches her ear is construed into a renewal of the first cause of her deep affliction; the gentlest wind which may happen to blow seems to threaten her yet more sorely."

"Every surrounding object appears, at length, tinctured with the cause of her misery; and each effort of herself and friends to shake off the horrid incubus is in vain."

"Time rolls on only to show how much she is the instrument of her involuntary feelings; and then the judgment is betrayed into acquiescence. She no longer merely feels her sufferings, but she seeks a cause for them; one which shall not only excuse them to herself, but be in strict harmony with her predominant feelings. And, thus, in passing from bad to worse, she realizes the precise condition of one labouring under *acute mania.*"

"The disease is, in such a case, *the necessary effect of an irritation of the ultimate structure of the brain;* and the consequence, only, of the application, through the medium of the external senses, of a stimulus so intense as to prove incompatible with the healthy physical capacities of the organ." . . . . "If such an abnormal state of the cerebral mass remains unrelieved, nothing is more likely than the occurrence of inflammation of the brain and its membranes, more or less insidious; and which progressing would necessarily induce those palpable disorganizations of structural effusions, &c., so generally observed."

"Such, I repeat it, are *generally* the *effects* of insanity, and not its *first cause.*"

"The patients in Hanwell are very liable to attacks of cerebral and meningeal inflammation, and which not unfrequently prove the immediate cause of death. In such cases the general symptoms which indicate the existence of inflammatory disease assume the same *asthenic* characters which belong to peripneumonia, euleritis, erysipelas, &c. &c., when occurring in *nervous* and *irritable* subjects. Upon the principle that such persons are more liable to the ordinary derangements of the general health, of which chronic inflammatory diseases form a great part, so are the insane *predisposed* to the occurrence of cerebral and meningeal inflammation, and *hence* the ordinary appearances observed after death."—*Zoist*, vol. i. p. 115, 116, 117. (1843.)

"The most appropriate and successful treatment consists in the administration of sedatives, with a generous diet, and the employment of those means calculated to improve the general health."

"Insanity, like other *nervous* diseases, is, invariably, aggravated by general bleeding."

"Many cases are cured in Hanwell by the use of wine and steel medicines." —*Zoist*, vol. i. p. 117. (1843.)

I feel, Sir, I need not make any remarks on the foregoing extracts: that they *settle* the question of PRIORITY, in so far as Dr. H. Munro and myself are concerned—to say nothing whatever of *Dr. Hitchman*—will be directly apparent to yourself and your numerous readers.

You will perceive, then, that my "*Nature and Proximate Cause of Insanity*" (which you have done me the honour to notice in your *valuable* journal—I quite mean this, in spite of my phrenological complainings,) is very like a SECOND EDITION of my paper "ON THE PATHOLOGY OF INSANITY," as contained in vol. i. of the *Zoist*, and dated July, 1833, (*more than ten years since.*)

To conclude, you write in the last No. of the PSYCHOLOGICAL—"*There is no difference between the theory of Munro and Davey.*" I would add, in all deference, there *is* just this dissimilarity—"Dr. H. Munro *limits the disease to* ONE *especial cause*," to quote your own words, whereas *I* maintain that "insanity is of *two* kinds, the one" (which is much the more common) "dependent on *nervous irritation* of the brain; and the other on *inflammation*, involving either the brain or its membranes."* The following few words were

* See *Zoist*, vol. i., 1843.

written in 1848, nearly three years before the publication of Dr. H. Munro's book, viz.:—"All those cases" (of insanity) "which owe their origin to a *physical* cause, are certainly inflammatory in their nature, and depend mainly on an increased vascularity of a particular portion or portions of the brain; but it is far otherwise with those cases of insanity induced by moral causes. If the disorder succeed to a severe and overpowering moral impression, to any great disappointment or alarm, or to outraged feeling of any kind, involving a sudden, unexpected, or violent shock of the nervous system, through the medium of any portion of cerebral matter, then are we disposed to attribute the phenomena observed *not* to inflammation, but to *nervous irritation* of the ultimate structure of the brain."*

In my *contributions to mental pathology*, published just one year before Dr. H. Munro's book, it is plainly seen that the foregoing opinions are much *exemplified* in certain parts,—*e. g.*, see pages 45, 97, 98, 100, 181, 192, 200, 201, 208, 219, 222, 223, 224, 225, 227, and 228.

The work of Dr. H. Munro, though professing to be written with *a specific object*,—viz., that of proving the atonic character of insanity, is, nevertheless, in good part, devoted to the consideration of other and extraneous questions,—viz., "the classification of the insane;" "arguments for the corporeal nature of insanity;" "the nature of ramolissement;" "the cause of general paralysis;" remarks on "Dr. Burnett's theory;" "statistics of Bethlem Hospital;" "note on phrenology," &c. &c.

The *theory*, therefore, constitutes rather the *ostensible* than the *real* basis of the volume; however, I have no wish to undervalue Dr. H. Munro's book; far from it.

I beg to remain, your obedient servant,

JAMES GEORGE DAVEY, M.D., &c. &c.

*Northwoods, Bristol, November 2nd,* 1853.

---

### A SUBJECT FOR PSYCHOLOGICAL ADJUDICATION.

* See my Contributions to Mental Pathology. 1850.

